# Evaluating the Impact of Aggregation Operators on Fuzzy Signatures for Robot Path Planning

**DOI:** 10.3390/s25237342

**Published:** 2025-12-02

**Authors:** Ahmet Mehmet Karadeniz, Csaba Hajdu, Áron Ballagi, László T. Kóczy

**Affiliations:** 1 Doctoral School of Multidisciplinary Engineering Sciences, Széchenyi István University, 9026 Györ, Hungary; 2 Department of Information Technology, Széchenyi István University, 9026 Györ, Hungary; hajdu.csaba@ga.sze.hu (C.H.); koczy@sze.hu (L.T.K.); 3 Department of Automation and Mechatronics, Széchenyi István University, 9026 Györ, Hungary; ballagi@sze.hu

**Keywords:** fuzzy signatures, aggregation operators, environment representation, mobile robots, A* algorithm, path planning

## Abstract

This study investigates the impact of different aggregation operators (commonly referred to as fuzzy operators) on the application of fuzzy signatures. Fuzzy signatures are specialized multidimensional data structures that symbolically represent data. As a use case, the study focuses on robot environment representation and path planning, presenting the results obtained by applying various aggregation operators including minimum, maximum, algebraic product and arithmetic mean on the normalized values obtained from the robot sensors. The comparison highlights their effects on the computational load and path lengths of the path planning task. The findings reveal that the most efficient aggregation operator, in terms of computational load and the path length, is the algebraic product aggregation operator. Specifically, the algebraic product consistently yielded the shortest paths (as low as 22 nodes) and the lowest execution times (down to 0.0913 s), demonstrating superior efficiency compared to the maximum operator, which resulted in path lengths up to 34 nodes and execution times reaching 0.1923 s. This represents an improvement of up to 35.3% reduction in path length and 52.5% reduction in execution time when comparing the algebraic product to the maximum operator based on observed extreme values. Furthermore, this work provides the first empirical comparison of fuzzy aggregation operators specifically for fuzzy signature-based mobile robot path planning.

## 1. Introduction

Research on environment representation plays a critical role in robotics, focusing on developing data structures that describe a robot’s surroundings, including obstacles and other detected features coming from the robot’s sensors. These structures are essential for algorithmic reasoning and tasks such as motion planning. Autonomous navigation in indoor environments, especially in the presence of static and dynamic obstacles, remains a major challenge for mobile robots. This area of research has produced various approaches to path optimization, with navigation in static environments being comparatively easier than in dynamic environments [1].

Different representation formats have been proposed, each tailored to specific scenarios. One widely used format is the occupancy grid-based representation, which discretely stores essential information [2]. Geometry-based formats, such as motion primitives, capture detailed object features and are supported by spatial data structures that index geometric information. Additionally, fuzzy inference systems provide a means of representing uncertain or imprecise information, with term descriptors, such as fuzzy signatures, structured in a nested format to enhance inference performance and to map hierarchically structured characteristics. Unlike traditional fuzzy inference systems, which often process data through a linear rule-base structure, FSigs offer a hierarchical framework for representing complex, multidimensional data and modeling interrelated subsets [3]. This hierarchical nested structure is a unique advantage, enabling symbolic reasoning across continuous data spaces and allowing complicated problems to be broken down into smaller, more manageable pieces, which is essential for structured environment representation.

Among existing environment representation formats, widely used occupancy grid-based representations, while discretely storing essential information, typically operate under the assumption of a perfectly known and static world. This assumption can render a pre-planned path invalid or dangerous in real-world robot navigation, which is characterized by significant uncertainty stemming from sensor noise, actuator errors, and the presence of dynamic obstacles. While fuzzy systems offer the ability to model a region’s ‘cost’ or ‘traversability’ as a fuzzy degree rather than a binary value (free/occupied), allowing for more nuanced decisions, research systematically utilizing fuzzy signatures (FSigs) as the primary data structure for environment representation is limited, and none specifically focuses on path planning tasks. Furthermore, in existing fuzzy signature applications, the choice of aggregation method is often based on theoretical convenience or lacks a comprehensive empirical analysis of its effect on key performance metrics, such as computational load and path optimization. Crucially, this foundational study is conducted under the strict assumption of a static environment to isolate the influence of the aggregation operators, thereby establishing a necessary baseline for future dynamic testing. Moreover, evidence from other domains shows that hierarchical aggregation of multidimensional sensor information can significantly improve task performance in complex systems, reinforcing the need for effective aggregation strategies in fuzzy signature–based environment representations [4]. This article directly addresses this gap. The primary contributions of this article are threefold:

1. It presents the first systematic application of fuzzy signatures (FSigs) as the primary environment representation data structure specifically for the mobile robot path planning task.
2. It provides a comprehensive empirical analysis comparing the effects of four fundamental aggregation operators (minimum, maximum, algebraic product and arithmetic mean) on the efficiency of the FSigs-based system.
3. It quantifies the impact of the chosen aggregation operator on key performance metrics, namely the resulting path length and the total computational execution time, offering evidence-based guidance for designing intelligent navigation systems. This work is the first empirical comparison of fuzzy aggregation operators specifically tailored for fuzzy signature-based mobile robot path planning.

This paper emphasizes the importance of selecting appropriate aggregation operators in fuzzy signatures by presenting the results of applying various operators to a path planning use case built on the novel environment representation method [5]. This method combines fuzzy signatures with quadtrees. The effects of each operator are analyzed in terms of path length and execution time and demonstrated through a detailed example of a reconstructed grid, which represents the output of the inference process based on the constructed nested structure.

The remainder of this paper is structured as follows: Section 2 reviews the related literature on Fuzzy Signatures, aggregation operators, and path planning in robotics, synthesizing the research gap. Section 3 details the methodology, covering Fuzzy Signatures, the four selected aggregation operators, the Fuzzy Situational Map/Quadtree environment representation method, and the A* path planning algorithm. Section 4 presents empirical results, comparing execution time and path length across the four operators. Finally, Section 5 concludes the study and proposes future research directions.

## 2. Related Works

This review employs a targeted, domain-bridging synthesis approach, structured to identify the critical intersection between fuzzy set theory and robotics. The methodology involves systematically analyzing the existing literature in three core, interconnected domains: the theoretical foundation and established applications of Fuzzy Signatures; the taxonomy and empirical usage of Aggregation Operators; and the state-of-the-art in mobile robot Path Planning. This structured review serves to synthesize findings and clearly delineate the specific research gap addressed by this study. This section reviews the key literature that forms the foundation of our study. We first examine the theoretical foundation and applications of fuzzy signatures and their associated aggregation operators. We then provide an overview of the path planning field in robotics, highlighting the challenges that motivate the use of intelligent, soft-computing approaches. Finally, we synthesize these two domains to identify the specific research gap this article addresses.

### 2.1. Application of Fuzzy Signatures and Aggregation Operators

The theoretical foundation of fuzzy signatures (FSigs) lies in Lotfi A. Zadeh’s pioneering work on fuzzy set theory [6], which researchers have expanded to develop structured ways of representing complex, multidimensional data. Building on this foundation, fuzzy signatures offer a powerful approach to knowledge representation, particularly for applications requiring symbolic reasoning across continuous data spaces [7]. What makes fuzzy signatures particularly valuable is their hierarchical structure, which enables researchers to break down complicated problems into smaller, more manageable pieces that can be addressed at different levels of detail.

This hierarchical approach gives fuzzy signatures a unique advantage when working with data that has complex structures, where individual features contain sub-features and exhibit complex relationships with one another. Researchers have successfully implemented fuzzy signatures in diverse fields, including medical diagnosis [8], image processing [9], and data mining [10], where the ability to capture and represent subtle and nuanced relationships between data elements becomes essential.

A critical operation in the utilization of fuzzy signatures is aggregation (see e.g., [11]). To derive a high-level decision or a lower-dimensional signature from a complex one, the values of its child branches must be combined. This is achieved using aggregation operators, known as a special type of fuzzy operators. The choice of operator is not arbitrary; it fundamentally shapes the behavior of the system. Common aggregation operators include:

- **T-norms (Triangular Norms):** Such as minimum and product. These operators are typically used for an “AND”-like aggregation, where the output is sensitive to the lowest input values. The minimum operator represents a pessimistic view (the chain is only as strong as its weakest link), while the product provides a smoother, more balanced aggregation.
- **T-conorms (Triangular Conorms):** Such as maximum. These are used for an “OR”-like aggregation, representing an optimistic view where the output is driven by the highest input value.
- **Averaging Operators:** Such as the arithmetic mean. These provide a neutral, linear combination of all input values.

The theoretical properties of these operators are well-established [12]. However, their empirical impact on the performance of a specific application, particularly in terms of computational efficiency and outcome quality, remains an area that requires further investigation.

### 2.2. Path Planning in Robotics

Path planning is a cornerstone problem in mobile robotics, involving the generation of a collision-free trajectory from a start configuration to a goal configuration. There is a vast body of work that can be broadly categorized. Classical methods include graph-search algorithms like $A^{*}$ [13] and Dijkstra’s algorithm, which are optimal in discrete, known environments [14]. The multi-robot task assignment problem, which often involves variants of the Traveling Salesman Problem (TSP) and Vehicle Routing Problem (VRP), is frequently classified as NP-hard, guiding researchers toward heuristic algorithms to find sub-optimal solutions due to the high computational burden required for optimal solutions in complex scenarios [15]. The challenge of covering defined 2D and 3D operational zones, known as Coverage Path Planning (CPP), often requires sophisticated extensions of classical algorithms or specialized metaheuristics to handle environments containing both static and dynamic obstacles, as demonstrated in numerous published studies.

Sampling-based methods, such as Probabilistic Roadmaps (PRM) and Rapidly exploring Random Trees (RRT), and their numerous modern variants, have been extensively reviewed for their effectiveness in finding feasible paths in high-dimensional, continuous spaces [16].

Despite their successes, these classical point-to-point search algorithms often operate under the assumption of a perfectly known and static world. Real-world robot navigation is characterized by significant uncertainty, stemming from sensor noise, actuator errors and the presence of dynamic obstacles [2]. This uncertainty can render a pre-planned path invalid or dangerous.

To address this challenge, researchers have turned to soft computing and artificial intelligence techniques [17]. Neural networks have been used for learning navigation behaviors and evolutionary algorithms have been applied to optimize paths in complex environments. Modern solutions often leverage Deep Reinforcement Learning (DRL), such as Multi-Agent Double Deep Q-Network (MA-DDQN) models, to determine optimal, energy-efficient trajectories and dynamic charging strategies, thereby minimizing task completion time for multi-UAV systems in complex, often NP-hard, monitoring environments [18].

Among these, fuzzy systems have emerged as a particularly powerful tool for its inherent ability to model and reason with vague information. Recent comprehensive reviews highlight the continued relevance and evolution of fuzzy systems in robotics and automation, demonstrating its robustness to sensor noise and its ability to generate smooth, human-like navigation behaviors for complex tasks [19]. The core idea is to represent the “cost” or “traversability” of a region not by a binary value (free/occupied) but as a fuzzy degree, allowing the robot to make more nuanced decisions.

Beyond mobile robot navigation, recent studies also show that fuzzy methodologies are increasingly being adopted in high-precision industrial applications such as robotic welding, where real-time vision-based systems leverage advanced image processing for seam tracking and control in dynamic environments [20]. This broader trend includes using fuzzy logic for efficient path planning and control in both static and dynamic environments, including algorithms like the Fuzzy Dynamic Window for local path planning [21] and utilizing ethologically inspired fuzzy behavior-based systems for robust navigation guidance [22].

### 2.3. Synthesis and Research Gap

Research systematically utilizing fuzzy signatures as the primary data structure for environment representation is limited, and no one specifically focuses on path planning tasks. More importantly, the impact of the choice of aggregation operator on the performance of such a system has been largely overlooked. In existing applications of fuzzy signatures, the aggregation method is often chosen based on theoretical convenience or without a comprehensive empirical analysis of its effect on key performance metrics like computational load and path optimization.

The systematic distinctions between existing fuzzy path planning methodologies, general Fuzzy Signature applications, and the novel approach presented here are systematically summarized in Table 1. We bridge the domain of fuzzy signatures with the practical challenge of robot path planning by constructing a navigation system based on a fuzzy signature representation of the environment. Subsequently, we conduct a systematic, empirical comparison of four fundamental aggregation operators, minimum, maximum, algebraic product and arithmetic mean, to quantify their specific effects on both the quality of the generated path (in terms of length) and the computational efficiency of the planning process. This provides the necessary evidence-based guidance for designing more effective and efficient intelligent navigation systems. Achieving such efficiency is essential, as demonstrated by studies that focus on rigorous performance evaluation, including the establishment of lower bounds on minimum service time, and the optimization of multi-agent systems to minimize overall mission or task completion time [23].

## 3. Methodology

In the following section, a thorough review of the research related to the proposed method is presented. This includes an in-depth exploration of the fundamental concepts of Fuzzy Signatures, Aggregation Operators, Fuzzy Situational Maps, Quadtrees and the $A^{*}$ path planning algorithm [29]. Furthermore, a detailed overview of the previously introduced environment representation [30] method is provided.

### 3.1. Fuzzy Signatures (FSigs)

**Figure 1:**
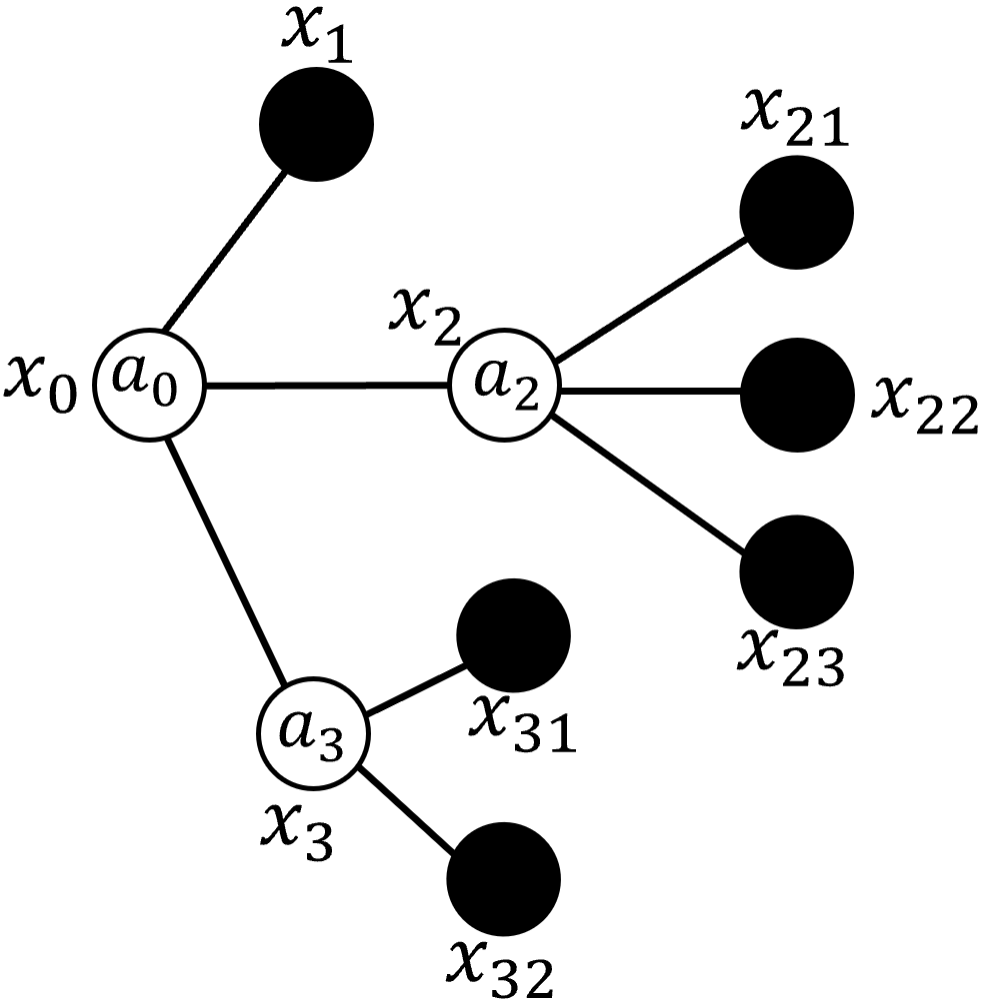
An example of a fuzzy signature graph, illustrating the fundamental nested hierarchical structure.

Fuzzy Signatures provide a hierarchical framework for representing multidimensional data with inherent uncertainties, enabling the effective modeling of interrelated subsets. These subsets collectively contribute to defining attributes of greater complexity or higher levels [27]. Figure 1 illustrates an example of the nested graph structure that is typical of a standard Fuzzy Signature.

There are two formats to represent Fuzzy Signatures: nested vectors and graphical structures [24]. The nested vector format corresponds to the hierarchical graph representation of Fuzzy Signatures, as illustrated in Figure 1, and is formally expressed in Equation (1). In this format, the first level is represented by the transposed vector ${\left[x_{1}\;x_{2}\;x_{3}\right]}^{T}$.

(1) $$x_{0}=a_{0}\left[\begin{array}{c} x_{1}\\ x_{2}\\ x_{3} \end{array}\right]=a_{0}\left[\begin{array}{c} x_{1}\\ a_{2}\left[\begin{array}{c} x_{21}\\ x_{22}\\ x_{23} \end{array}\right]\\ a_{3}\left[\begin{array}{c} x_{31}\\ x_{32} \end{array}\right] \end{array}\right]$$

The structure of fuzzy signatures, as outlined in Equation (1), demonstrates how the membership degrees assigned to $\left[x_{21}\;x_{22}\;x_{23}\right]$ are combined using the aggregation operator $a_{2}$ to produce the degree of membership assigned to the component $x_{2}$, while the membership degrees of the elements $\left[x_{31}\;x_{32}\right]$ are combined using the aggregation operator $a_{3}$ to form the membership degree assigned to $x_{3}$. These membership degrees are integrated through specific fuzzy aggregation operators, whose selection is crucial for effectively combining data within the fuzzy signature framework. This is particularly important in scenarios where certain variable details (represented as graph nodes or leaf nodes) may be incomplete or missing within subsets. Commonly used aggregation operators, in addition to the weighted generalized mean, include the functions min and max, which serve as the fundamental t-norm and s-norm operators, respectively [7].

A numerical example of the fuzzy signature discussed is provided in Equation (2) to further clarify this concept. In this example, it is assumed that the aggregation operator max is applied at deeper levels, while the aggregation operator min is utilized at the first level. Aggregation operators are explained in detail in Section 3.2.

(2) $$x_{0}=min\left[\begin{array}{c} 0.9\\ max\left[\begin{array}{c} 0.5\\ 0.7\\ 0.4 \end{array}\right]\\ max\left[\begin{array}{c} 0.6\\ 0.8 \end{array}\right] \end{array}\right]$$

The first level of the fuzzy signature hierarchy can be computed as demonstrated in Equation (3), by employing the aggregation operator max at the respective branches:

(3) $$x_{0}=min\left[\begin{array}{c} 0.9\\ 0.7\\ 0.8 \end{array}\right]\Rightarrow \left[\begin{array}{c} 0.7 \end{array}\right]$$

**Figure 2:**
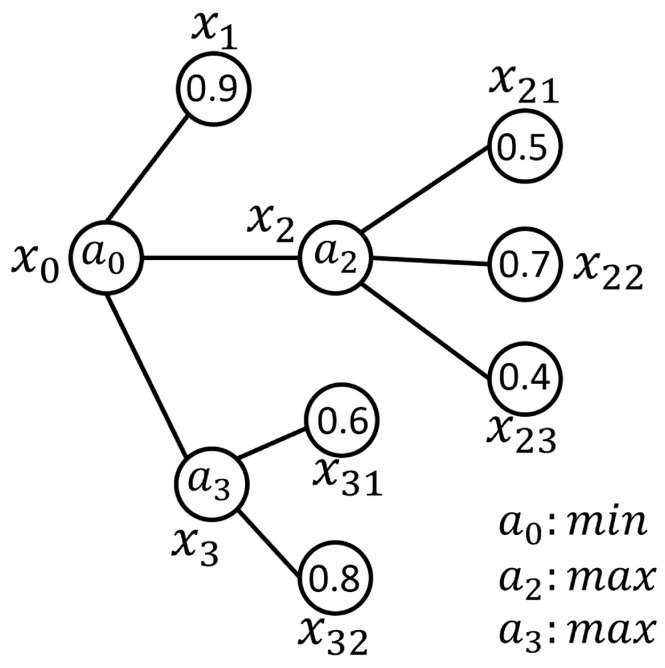
Graph representation of the numerical fuzzy signature example, detailing the assignment and aggregation of membership degrees.

The graph representation of the provided numerical example, including the membership degrees on the branches, is shown in Figure 2.

In the graph representation of the fuzzy signatures depicted in Figure 2, the aggregation operators are represented as $a_{0}$, $a_{2}$, and $a_{3}$. Specifically, the operator $a_{2}$ combines the membership degrees assigned to the elements $x_{21}$, $x_{22}$, and $x_{23}$ to produce the membership degree assigned to $x_{2}$, while $a_{3}$ combines the membership degrees assigned to $x_{31}$ and $x_{32}$ to compute $x_{3}$. Both $a_{2}$ and $a_{3}$ employ the aggregation operator max.

**Figure 3:**
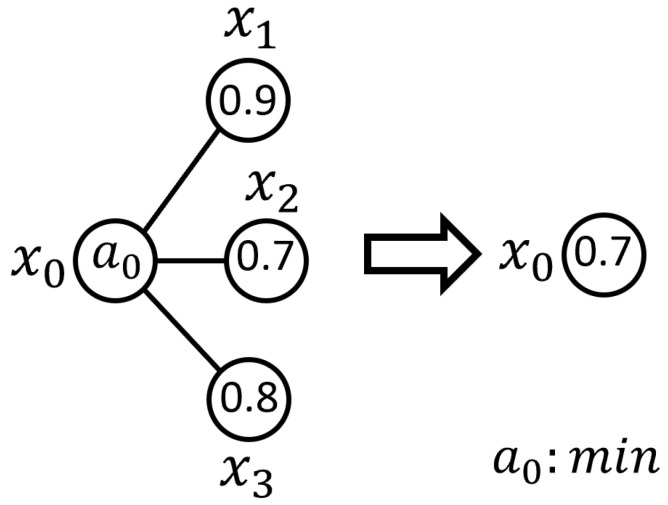
The obtained root-level graph representation, showing the resulting membership degree $x_{0}$ after hierarchical aggregation.

The values for the higher levels can be systematically derived by applying these fuzzy aggregation processes to the deeper-level components. This hierarchical computation ultimately leads to the root-level representation of the fuzzy signature graph, as illustrated in Figure 3. At this level, the operator $a_{0}$—defined as the aggregation operator min—calculates the final root-level value, representing the resulting membership degree assigned to $x_{0}$ of the fuzzy signature graph.

### 3.2. Aggregation Operators and Assessment of Their Impacts

A Fuzzy Signature employs aggregation operator to combine the membership degrees of its branches. These aggregation operators at different branches can be all different types of as well as same kind. In this study, the obtained Fuzzy Signature structure has two-level, and each chosen aggregation operator is used at both levels separately. The path planning use case has been employed to assess the impacts of each aggregation operator on the robot environment representation Fuzzy Signature. The selection of these specific four operators (minimum, maximum, algebraic product, and arithmetic mean) was deliberate, as they represent the fundamental and distinct categories of fuzzy aggregation techniques: T-norms (min, product), T-conorms (max), and averaging operators (mean). This systematic empirical comparison focuses on quantifying the performance variability across these core operational extremes and central tendencies. The four aggregation operators utilized in this study are formally defined in Equations (4)–(7) based on established fuzzy systems literature:

- **The Minimum Aggregation Operator (T-norm):** This operator returns the minimum of the *k* input membership values ($\mu_{i1},\ldots ,\mu_{ik}$), as defined in Equation (4) [31].
  (4) $$\mu_{i}=\min\left\{\mu_{i1},\mu_{i2},\ldots ,\mu_{ik}\right\}\;$$
- **The Maximum Aggregation Operator (T-conorm):** This operator returns the maximum of the *k* input membership values, as defined in Equation (5) [31].
  (5) $$\mu_{i}=\max\left\{\mu_{i1},\mu_{i2},\ldots ,\mu_{ik}\right\}\;$$
- **The Algebraic Product Aggregation Operator (T-norm):** This operator yields the product of the *k* input membership values, as defined in Equation (6) [31].
  (6) $$\mu_{i}=\prod_{n=1}^{k}\mu_{ik}\;$$
- **The Arithmetic Mean Aggregation Operator (Averaging):** This averaging operator outputs the mean of the *k* input membership values, as defined in Equation (7) [31].
  (7) $$\mu_{i}=\frac{\mu_{i1}+\mu_{i2}+\cdots +\mu_{ik}}{k}\;$$

### 3.3. Fuzzy Situational Maps (FSMs)

Fuzzy situational maps, modeled as multidimensional extended FSigs, are well-suited for representing complex multidimensional system conditions, particularly when the information is incomplete, distorted, or noisy. The values within individual nodes can be interpreted as components of a fuzzy signature, making Fuzzy Situational Maps describable as multidimensional, spatially organized fuzzy signatures.

Each node in a Fuzzy Situational Map (FSM) can itself be a nested FSM and this process can be repeated iteratively, allowing for increased depth (finer resolution). This approach results in a finely structured FSM within each node. Notably, the resolution of each node operates independently of others. This operation of extending the depth of an FSM is referred to as a “zoom-in” process [28].

The individual nodes and their associated sub-lattices (high-resolution lattices) are interconnected, as the subgroups of sub-lattices collectively define the characteristics of the higher (parent) level. This structure allows each node to store a substantial amount of additional information, which is processed only at the required resolution depth, significantly reducing computational demands. Fuzzy situational maps (FSM) offer a more efficient way to represent hierarchically structured multidimensional data compared to traditional fuzzy signatures.

An additional advantage of FSMs is their ability not only to organize external information in a hierarchical structure but also to account for the interactions between individual nodes. This means that each node is connected to its neighboring nodes and changes in its value can influence those neighbors. These interactions can be either unidirectional or bidirectional and may impact on the entire situational map or only specific sub-lattices within the map.

FSMs also provide natural mechanisms for handling temporal dynamics by updating fuzzy membership values as new information (such as new sensor observations) become available. This temporal aggregation capability makes FSMs particularly suitable for applications involving dynamic environments where it is essential to integrate new information while preserving historical confidence levels.

### 3.4. Quadtrees

Hierarchical spatial data structures known as quadtrees [32] provide an efficient approach for representing two-dimensional spatial information. These tree-based structures have demonstrated significant utility across various fields including image processing and geometric reconstruction, offering the typical benefits associated with hierarchical data organization while enabling rapid execution of spatial queries such as range and point location operations. A distinguishing characteristic of quadtrees lies in their systematic branching pattern, where each internal node invariably subdivides into exactly four offspring nodes. The operational domain of quadtrees is typically constrained to a finite spatial region that undergoes recursive subdivision into progressively smaller areas. This hierarchical partitioning concept can be extended to three-dimensional applications, creating structures termed octrees [33], wherein each node generates eight child nodes. Beyond spatial modeling, the octree structure is also widely applied in other domains such as image processing, where it forms the basis for vector quantization and hierarchical clustering techniques [34].

**Figure 4:**
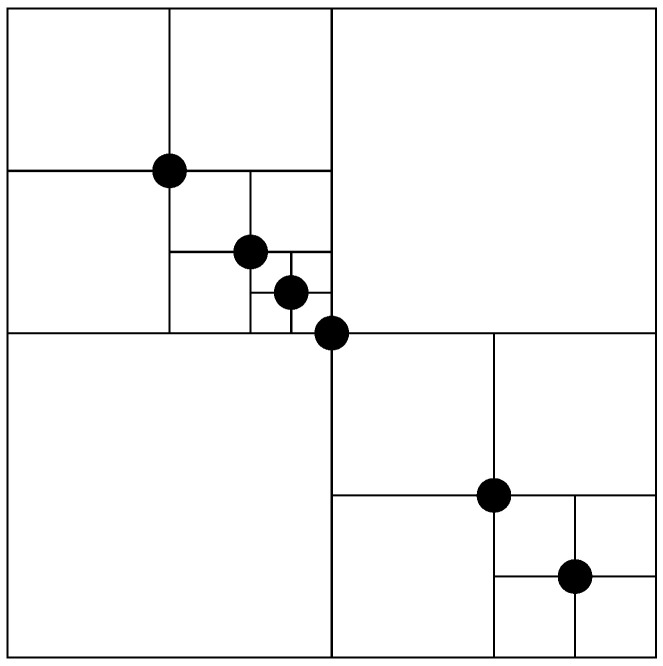
An illustration of a Quadtree structure demonstrating adaptive spatial subdivision based on inserted points [24].

The core algorithmic methodology centers on the systematic partitioning of spatial regions into discrete subdivisions. Point assignment to specific subdivisions is determined by evaluating the spatial relationship between the point coordinates and the subdivision’s reference origin, particularly when incorporating new data points. Subsequent points undergo systematic placement into appropriate subsections based on their positional coordinates. This iterative insertion procedure continues until reaching a predetermined subdivision depth within the hierarchical structure. In contrast to conventional quadtrees that impose uniform spatial partitioning, Figure 4 demonstrates a quadtree constructed from a specific point distribution, clearly illustrating the adaptive spatial subdivision based on the actual data point locations.

Consider a quadtree structure where individual nodes represent discrete spatial regions. Initially, the quadtree exists as an empty structure containing a single root node that encompasses the entire spatial domain. Through the sequential insertion of data points, the quadtree dynamically subdivides the spatial domain into progressively finer partitions.

The graphical representation presented in Figure 4 displays a hierarchy of nested rectangular regions. Each rectangular area undergoes further subdivision into four smaller quadrants, with data points (represented as dots) positioned within the finest level subdivisions.

### 3.5. Fuzzy Signatures Based Environment Representation

The method integrates spatial data structures for representing the environment of the robot, specifically Quadtrees-like structure with fuzzy system-based techniques to create a robust and comprehensive framework for representing environmental characteristics. This approach is inspired by FSMs [28], which divide the robot’s workspace into areas of varying levels of interest and granularity. The primary objective is to systematically identify obstacle points based on sensory inputs and assign them fuzzy values derived from rule sets that assess the importance or significance of each point.

**Figure 5:**
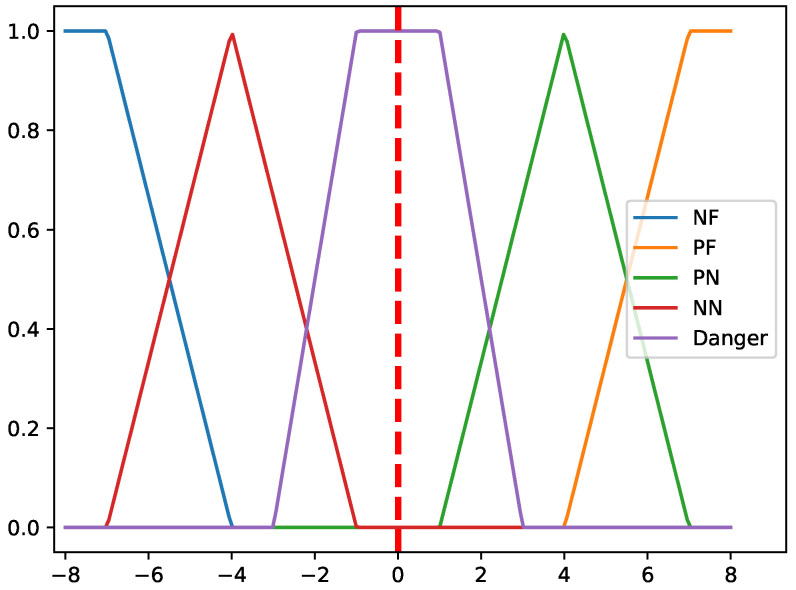
Associated fuzzy membership functions (NF, NN, D, PN, PF) used for translating normalized sensor data into traversability costs.

The membership functions utilized, as visualized in Figure 5, are predominantly triangular and trapezoidal in form. This structure was specifically chosen for its computational simplicity and effective representation of overlapping linguistic concepts (NF, NN, D, PN, PF). These five fuzzy sets span the normalized sensory space (the X and Y axes) relative to the robot’s starting position (0,0). Crucially, the parameters defining these functions—including the center points, spread, and boundary coordinates—are directly calibrated based on the physical and operational parameters of the robot’s sensors. For example, the range of the ‘Danger (D)’ set is meticulously aligned with the robot’s minimum safe stopping distance or collision threshold determined by its proximity sensor characteristics, while the boundaries of the ‘Near’ (NN, PN) and ‘Far’ (NF, PF) sets correspond to the maximum effective sensing range and necessary planning horizons. This correlation ensures that the fuzzy membership degree assigned to an environmental characteristic accurately translates the raw sensor distance data into a quantifiable measure of risk or traversability cost.

With the set of membership functions illustrated in Figure 5 fuzzy operations (aggregation operators) are defined over $F_{x}$ and $F_{y}$ to transform uncertain node coordinates into fuzzy values along each axis. The product aggregation operator is employed for both axes in this study. Each fuzzy set conveys a distinct meaning, representing various characteristics of the environment or the significance of the spatial data along the *X* and *Y* axes:

- *Negative Far (NF):* Indicates a situation where the attribute of interest is positioned far away from the robot on the negative side of the axis, signifying no immediate threat or concern.
- *Negative Near (NN):* Represents that the attribute is relatively near to the robot on the negative side of the axis, posing a significant risk that requires attention.
- *Danger (D):* Denotes a critical scenario where the attribute is dangerously close to the robot, necessitating immediate action to avoid a potential collision.
- *Positive Near (PN):* Similar to NN, but on the positive side of the axis, signifying a considerable risk due to proximity to the robot.
- *Positive Far (PF):* Similar to NF, but on the positive side of the axis, indicating that the attribute is far from the robot and does not pose an immediate danger.

These interpretations play a crucial role in assessing the proximity and potential threat posed by various attributes in the environment of the robot [24].

**Figure 6:**
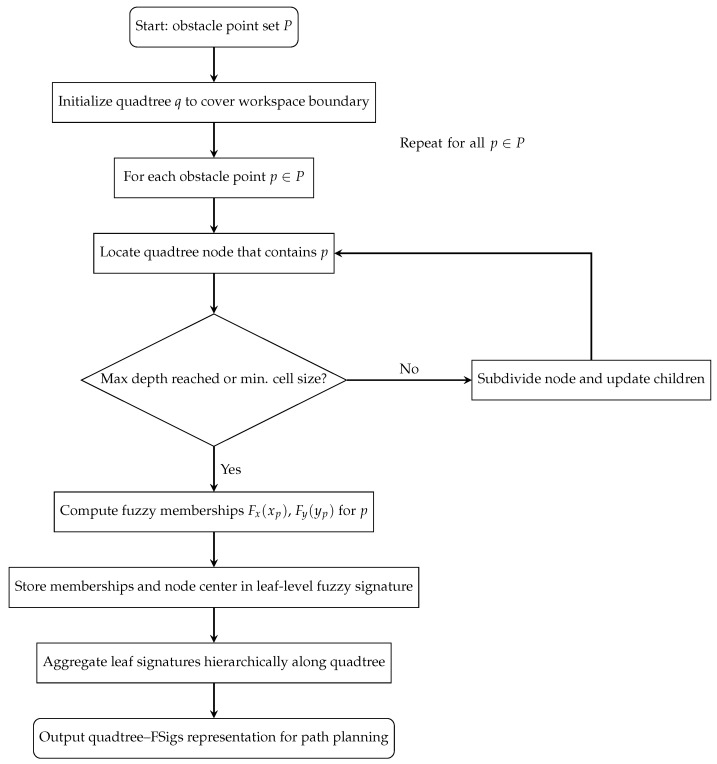
Flowchart summarizing the construction of the Quadtree-FSig representation. Starting from the obstacle point set *P*, the environment is incrementally subdivided into quadtree cells, fuzzy memberships are computed for each obstacle point at the leaf nodes, and the resulting leaf-level fuzzy signatures are hierarchically aggregated to form the final environment representation used for path planning.

In order to make the construction of the quadtree–fuzzy signature representation more transparent, the overall procedure is summarized in the flowchart in Figure 6. Starting from the set of obstacle points *P*, an initial quadtree *q* is first defined to cover the entire boundary area. Then, for each obstacle point p∈P, the corresponding quadtree node is located. If the node has not yet reached the maximum depth or minimum allowable cell size, it is subdivided and its children are updated accordingly. Otherwise, the fuzzy membership degrees $F_{x}\left(x_{p}\right)$ and $F_{y}\left(y_{p}\right)$ are computed and stored in the leaf-level fuzzy signature together with the node’s center coordinates. After processing each point, the procedure loops back to evaluate the next obstacle point, ensuring a consistent top-down traversal of the dataset. Once all points have been processed, the leaf-level fuzzy signatures are hierarchically aggregated upward through the quadtree to form the final environment representation used for path planning.

The process for constructing an environmental representation based on a set of two-dimensional obstacle points, denoted as *P*, is described in the following steps:

1. Begin by setting up the quadtree *q* to cover the designated boundary area of the robot’s environment. This boundary defines the spatial extent within which the robot operates and where obstacles may be present.
2. For each point *p* in the set *P*, divide the space as needed and place *p* at the center of the newly formed sector. Identify the fuzzy sets associated with *p* and save them alongside their coordinates. Modify the membership function of the obstacle to represent its specific properties. Create four child nodes for each node, defining their subdivided boundaries in advance but leaving their values unassigned for now.
3. When a sector reaches its maximum allowable depth, then update the fuzzy signature value associated with that node. Modify the membership functions if necessary, which might happen when a new obstacle with more significant attributes is identified or because of the defuzzification process.

During the construction of the quadtree, each node is defined by a 4-tuple consisting of $\left(x,y,F_{x}\left(x\right),F_{y}\left(y\right)\right)$. This tuple includes the spatial coordinates (x,y) and the fuzzy sets $\left(F_{x}\left(x\right),F_{y}\left(y\right)\right)$, which are evaluated along the *X* and *Y* axes, respectively. Optionally, the results of the defuzzification process can also be stored within each node to aid in decision-making processes. Additionally, nodes may contain details about the membership functions and their parameters, further enhancing the inference capabilities of the framework.

In this context, the starting point of the quadtree-like structure is always aligned with the central position of the robot, ensuring a reference point for representing the surrounding environment.

By following these steps, the quadtree-like structure is filled with nodes that capture the spatial distribution of obstacles, where each node contains fuzzy values providing a detailed depiction of environmental characteristics in relation to the position of the robot.

**Figure 7:**
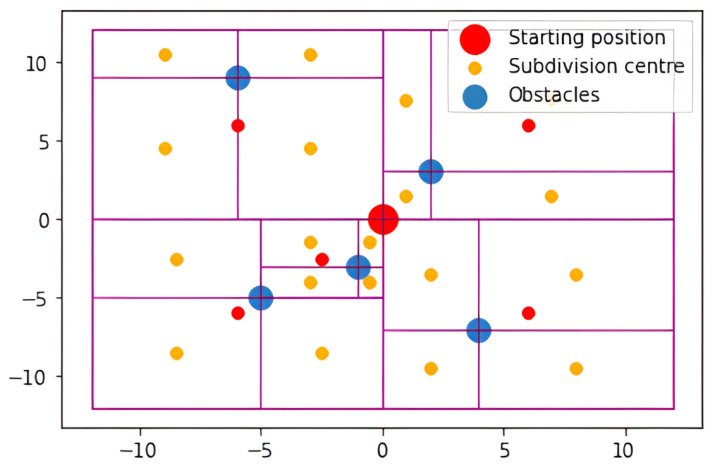
Visualization of quadtree-like FSM subdivisions for representing the environment. The robot’s starting position is marked by a red circle, obstacle points are indicated by blue circles, orange circles denote the centers of the leaf-node subdivisions, and purple rectangles outline the boundaries corresponding to each depth level within the quadtree-like structure.

Figure 7 showcases a typical example of a quadtree-like Fuzzy Situational Map (FSM) created using the described method. The hierarchical structure of the representation can be utilized during the inferential process. For instance, when constructing a grid, each point can be assessed through the defuzzification process applied to the FSM. This allows for the extraction of precise, actionable insights from the fuzzy environmental model, supporting decision-making tasks such as navigation [25,26] or obstacle avoidance [22] based on sensory information in robotic or autonomous systems.

Building on the previously discussed methodologies and the introduction of this novel concept, combining fuzzy signatures with a quadtree-based environmental representation offers a promising approach that effectively balances computational efficiency and dimensionality management.

This method enables the creation of progressively detailed grids, refining the grid until the required resolution is achieved to detect each object in the mobile robot’s surroundings. The grid construction process is intricate, involving the integration of fuzzy signatures and quadtrees. The environment is subdivided into four sections using quadtrees, and the resulting data is stored within fuzzy signatures. This iterative process continues until all objects are identified, ensuring thorough coverage and a detailed representation of the environment.

The proposed representation, although inspired by FSMs which are also based on extended fuzzy signatures, fundamentally differs in its systematic enforcement of spatial hierarchy. While generic FSMs define a hierarchical structure where the resolution of each node can operate independently during the ‘zoom-in’ process, our methodology strictly adheres to the rigid, systematic branching pattern of the Quadtree structure. Specifically, the Quadtree mechanism is utilized to enforce a standardized spatial decomposition, where every internal node invariably subdivides into exactly four offspring nodes. This approach tightly couples the fuzzy signature’s hierarchical definition with a standardized geometric partitioning, thereby creating a representation optimized for rapid spatial queries and consistent dimensionality management in robotics path planning applications.

**Figure 8:**
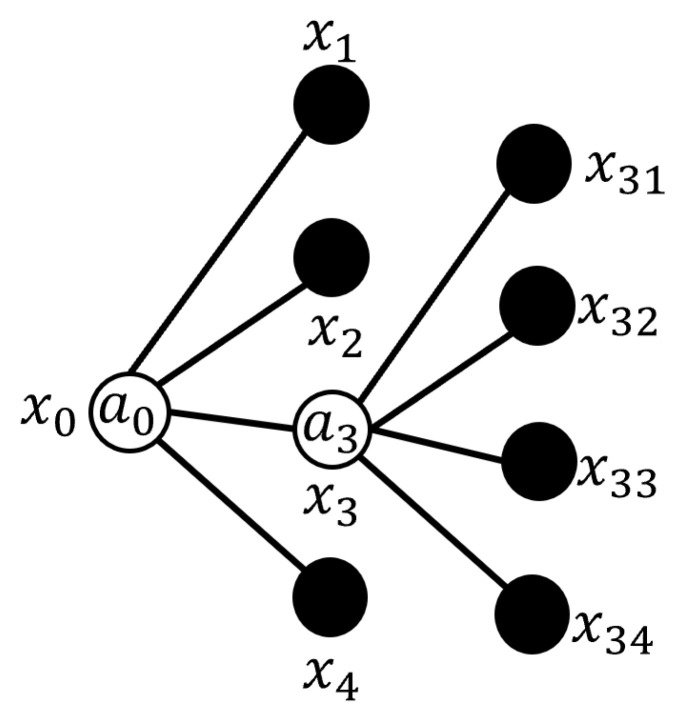
The hierarchical fuzzy signature graph corresponding to the robot environment representation, illustrating the spatial partitioning and aggregation points.

For example, the fuzzy signature graph corresponding to the scenario depicted in Figure 7 is presented in Figure 8. In this graph, $x_{1}$ represents the top-right section, while $x_{2}$, $x_{3}$, and $x_{4}$ correspond to the subsequent sections, arranged counterclockwise starting from $x_{1}$. Furthermore, $x_{31}$, $x_{32}$, $x_{33}$, and $x_{34}$ represent the subsections of section $x_{3}$. The presence of closer obstacles in section $x_{3}$ necessitates a higher resolution to capture more detailed information about its subsections. The aggregation operators used are represented by $a_{3}$ and $a_{0}$ to combine the membership degrees assigned to $x_{3}$ and $x_{0}$, respectively. This visual representation effectively illustrates how the environment is partitioned and represented, as well as how obstacle position information is stored, showcasing the efficiency and utility of the proposed approach.

### 3.6. $A^{*}$ Path Planning Algorithm

The $A^{*}$ algorithm, first introduced by Hart et al. [14], is a grid-based approach designed to identify the shortest path for a given problem. It is widely used to determine the most cost-effective route from a starting point to a target destination. Known for its high efficiency and low computational overhead, $A^{*}$ has become a powerful pathfinding tool in various fields, including satellite navigation for road vehicles and routing strategies for robots and autonomous systems [35]. The algorithm operates by calculating the cost $f(N_{i})$ for reaching all neighboring nodes, where $N_{i}$ are adjacent nodes. The primary objective of $A^{*}$ is to advance toward the node with the lowest $f(N_{i})$ value, which is determined using the formula presented in Equation (8):

(8) $$f\left(N_{i}\right)=g\left(N_{i}\right)+h\left(N_{i}\right)$$

In this equation, $g(N_{i})$ represents the actual distance or cost from the starting node to the current node $N_{i}$, while $h(N_{i})$ denotes the heuristic estimate of the cost to reach the destination from node $N_{i}$. Consequently, the algorithm prioritizes expanding the node with the smallest $f(N_{i})$ value during each iteration of the search process.

In the context of this fuzzy path planning application, the $g(N_{i})$ cost component is directly adapted by the fuzzy environment representation. The environment is represented as a grid whose costs are derived from the defuzzified output of the Fuzzy Situational Map (FSM). Specifically, the membership values, determined by the chosen aggregation operator (min, max, algebraic product, or arithmetic mean), dictate the localized traversability cost. A higher defuzzified value corresponding to an obstacle region translates into a proportional increase in the movement cost $g(N_{i})$, effectively integrating the fuzzy risk directly into the $A^{*}$ cost function. Regarding the heuristic $h(N_{i})$, this study utilizes the standard Euclidean distance between the current node $N_{i}$ and the goal position as the heuristic function. This non-fuzzy, admissible heuristic ensures efficient search while the fuzzy membership degree (via the FSM) modulates the actual cumulative cost $g(N_{i})$.

The steps of the $A^{*}$ graph-based pathfinding algorithm are outlined in Algorithm 1.
**Algorithm 1** $A^{*}$ Path Finding Algorithm1:Initialize open list with Start node, closed list as empty2:**while** open list is not empty **do**3:   Take node with lowest *f* from open list4:   **if** node is Goal **then**5:      Reconstruct path from Start to Goal6:      **return** Path7:   **end if**8:   Move node to closed list9:   **for** each neighbor of node **do**10:      Calculate *g*, *h*, and *f* for neighbor11:      **if** neighbor is not in open list with lower *f* **then**12:          Update neighbor’s parent, *g*, *h*, and *f*13:          Add neighbor to open list if not present14:      **end if**15:   **end for**16:**end while**17:**return** Failure

## 4. Results

The goal of this study is to explore how different fuzzy aggregation operators (min, max, prod, mean) affect the path planning results in an environment with obstacles. Each of the chosen aggregation operators is employed in the Fuzzy Signature structure separately and their results are presented. To compare the results, two parameters were chosen in this study: the resulting path length and the execution time.

As shown in Figure 7, the robot is in coordinates (0, 0). Therefore, the starting point is determined as the robot’s position. For the purposes of this foundational comparison, which aimed to establish the general performance characteristics of the aggregation operators, extreme points were tried to be chosen to have a better overview about the comparison between aggregation operators in the path-planning use case. Hence, (−10, −10), (10, −10), (10, 10) and (−10, 10) points are chosen as goal positions. Crucially, these initial experiments were intentionally restricted to static environments to isolate and quantify the fundamental impact of the aggregation operator choice on the $A^{*}$ search efficiency without the confusing variables introduced by temporal dynamics. The path length and execution time are then recorded for each of these goal points. The results presented in Table 2 represent the average execution time calculated from 10 independent simulation runs for each combination of aggregation operator and goal position. This averaging procedure was crucial for mitigating noise measurement and random variability introduced by operating system overhead. The simulation environment was implemented in python programming language and executed on a PC equipped with Intel Core Ultra 5 and 16 GB RAM.

**Figure 9:**
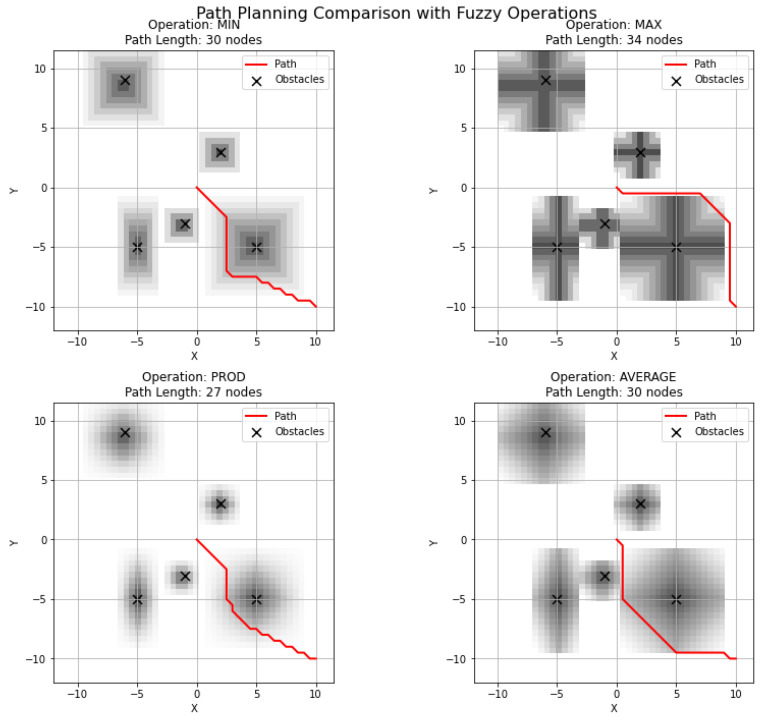
An example of the comparison of aggregation operators in terms of path lengths for the goal position (10, −10).

An example of a comparison of the path lengths resulting for the aggregation operators chosen is shown in Figure 9. It can be seen that the shortest path length occurred when the algebraic product aggregation operator is used.

The overall results obtained in this study employing four distinct aggregation operators individually for the path planning use case are presented in Table 2.

## 5. Discussion

### 5.1. Theoretical Computational Complexity Comparison

The overall computational complexity of the environment representation process is dominated by the construction of the Quadtree-like FSM structure, which is independent of the choice of aggregation operator. This hierarchical method ensures that detailed information is processed only at the necessary resolution depth, leading to significantly reduced computational demands compared to flat grid structures.

The complexity difference among the four operators arises in the constant time factor associated with Step 3 of the construction process (updating the fuzzy signature value) and, more importantly, during the subsequent path planning step ($A^{*}$ traversal), where the aggregation operator determines the “traversability” cost of the path.

When aggregating k input membership values $\left(\mu_{i1},\ldots ,\mu_{ik}\right)$ at a single node, the fundamental operational differences are:

For minimum(MIN) and maximum(MAX) operators, defined in Equations (4) and (5), k−1 comparison operations are required. Comparisons are typically considered the simplest and fastest fundamental computational operations. The MIN operator is interpreted as a T-norm (pessimistic) while the MAX is as a T-conorm (optimistic).

For algebraic product(PROD) operator is defined by multiplication as shown in Equation (6) and k−1 multiplication operations are required. Multiplication is generally more resource-intensive than comparison or addition.

Arithmetic Mean(MEAN) operator is defined by addition and division as shown in Equation (7). It requires k−1 additional operations followed by one division operation. Division operations are generally the most complex and time-consuming of these fundamental mathematical operations.

Based purely on the constant-time operation count at a single node, MIN and MAX are theoretically the fastest, followed by PROD, and MEAN is often the slowest due to the division requirement.

However, the empirical results shown in Table 2 demonstrate that the algebraic product operator (PROD) yields the lowest execution time, followed closely by MIN. This apparent contradiction—where an operator utilizing slower multiplication operations performs better than those relying on faster comparisons (MIN/MAX)—highlights that the computational efficiency is dictated not by the cost of the aggregation operation itself, but by the qualitative nature of the resulting fuzzy value, which significantly impacts the $A^{*}$ search efficiency. Specifically, the algebraic product is described as providing a smoother, more balanced aggregation compared to the minimum operator. Because the algebraic product is a T-norm based on multiplication, it generates a representation where the cumulative membership degree rapidly decreases (i.e., penalizes traversability) when multiple underlying factors (Fuzzy Signature components) indicate a potential obstacle or complexity, even if none of the individual factors represent an extreme danger.

This characteristic of the algebraic product, often interpreted as generating smoother gradients in the cost surface, creates a highly differentiated and well-defined cost landscape for the $A^{*}$ algorithm. This allows the $A^{*}$ heuristic to more effectively prune inefficient search branches because the cost estimate ($f(N_{i})$) accurately reflects the cumulative risk of traversing a complex region. The result is that the $A^{*}$ algorithm explores fewer nodes (as evidenced by PROD consistently yielding the shortest path length in nodes across all tests) before finding the goal, thus minimizing the total computational overhead of the search process. This reduction in the overall number of required $A^{*}$ operations decisively outweighs the slightly higher constant-time cost of the multiplication operation compared to comparison or min/max operations.

The algebraic product, being a smoother aggregation, likely generates a grid representation that allows the $A^{*}$ algorithm to explore fewer nodes before finding the goal (as evidenced by PROD yielding the shortest path length in nodes across all tests), thus minimizing the total computational overhead of the search process. This reduction in the overall number of required $A^{*}$ operations outweighs the slightly higher constant-time cost of the multiplication operation compared to comparison or min/max operations.

### 5.2. Practical Implications and Generalizability

The findings underscore the importance of selecting appropriate aggregation operators, as they significantly impact the computational load and the efficiency of the fuzzy signatures-based approach. For the path planning use case, the algebraic product aggregation operator resulted in the shortest path length and the lowest computational load. These results highlight the need for careful consideration of aggregation operators based on the specific requirements of a given application.

This efficiency gain, particularly the 52.5% reduction in execution time and 35.3% reduction in path length observed when using the algebraic product compared to the maximum operator, is crucial for resource-constrained embedded systems and actual mobile robots, where minimal latency and reduced energy consumption are paramount for robust real-time navigation. This established performance metric, demonstrating superior computational efficiency and path optimization, constitutes the primary Practical Managerial Significance (PMS) of the proposal, providing reliable, evidence-based guidance for deployment in resource-constrained embedded systems and real-world autonomous robotic platforms.

Furthermore, the inherent hierarchical and multidimensional nature of Fuzzy Signatures (FSigs) ensures the generalizability of this optimized aggregation approach beyond simple point-to-point navigation, making the findings directly relevant for complex applications such as large-scale Coverage Path Planning (CPP) and multi-agent coordination, where efficiency is paramount, and also allowing for application in diverse domains, as evidenced by previous FSig usage in medical diagnosis [8] and data mining [10]. This research on path planning demonstrates the versatility and applicability of fuzzy signatures across various domains. By tailoring their use to the specific requirements of a given task, fuzzy signatures can effectively address the needs.

### 5.3. Future Work

Future research could explore additional aggregation operators, comparing new and existing ones across various scenarios to enhance the applicability of fuzzy signatures in path planning tasks [36]. Another possibility could involve investigating the use of metaheuristic or reinforcement learning (RL) techniques with the fuzzy signature structure during the aggregation process to identify the optimal aggregation operator for each specific goal position. Crucially, extensions to tasks such as Coverage Path Planning (CPP) and multi-agent coordination, which inherently involve dynamic and complex operational zones, are a logical next step. Furthermore, a crucial area for future investigation involves systematically analyzing the sensitivity of the path planning performance to the structural parameters of the environment representation, specifically the effect of varying the quadtree depth (resolution) and the impact of alternative designs or parameters for the associated fuzzy membership functions (Fx(x), Fy(y), as defined in the methodology). This analysis must specifically evaluate whether changes in the membership function shapes (e.g., transitioning from triangular to Gaussian functions) or their scaling (e.g., adjusting the center points or spreads of the NF, NN, D, PN, and PF sets) alter the observed performance ranking of the distinct aggregation operators. Crucially, future empirical work must also include testing intermediate goal coordinates and targets located within or adjacent to obstacle-dense regions of the map, as this will determine if the observed efficiency ranking of the aggregation operators (particularly the *algebraic* product) remains consistent when the path planning search space involves maximal localized complexity and variable quadtree resolution levels.

Finally, given that this study utilizes static obstacles, future research must address dynamic environments. The FSMs utilized here are well-suited for information that is incomplete, distorted, or noisy, and possess natural mechanisms for handling temporal dynamics by updating fuzzy membership values as new sensor observations become available. Therefore, a critical extension involves testing the robustness of the chosen aggregation operators against moving obstacles and time-varying uncertainty, leveraging the FSM’s temporal aggregation capability to integrate new information while preserving historical confidence levels.

## 6. Conclusions

This study systematically evaluated the influence of four widely used aggregation operators—minimum, maximum, algebraic product and arithmetic mean—on the performance of a fuzzy-signature-based environment representation applied to robot path planning. Using a consistent hierarchical structure integrating fuzzy signatures with a quadtree-based spatial model, each operator was independently incorporated into the inference process, after which the resulting environment representation was processed by the $A^{*}$ algorithm [37]. The comparative analysis focused on two key performance indicators: the total path length and the execution time required to compute a valid path.

The results reveal that the choice of aggregation operator has a pronounced impact on the efficiency of the planning process. Among the evaluated operators, the algebraic product consistently exhibited superior performance, yielding both the shortest paths (as low as 22 nodes) and the lowest execution times (down to 0.0913 s). Conversely, the maximum operator produced the longest paths (up to 34 nodes) and the highest execution times (up to 0.1923 s). These findings correspond to an improvement of 35.3% in path length and 52.5% in execution time in favor of the algebraic product operator when compared with the maximum. Intermediate performance characteristics were observed for the minimum and arithmetic mean operators.

Overall, the empirical evidence demonstrates that aggregation operators generating more discriminative and smoothly varying cost representations—such as the algebraic product—facilitate more efficient exploration within the $A^{*}$ search process. Despite all operators operating on the same fuzzy signature structure, their different aggregation behaviors significantly influence the resulting traversability map and, consequently, the global characteristics of the search. These results highlight the critical role of aggregation operator selection in fuzzy-signature-based navigation frameworks and underline its direct impact on computational efficiency and path-planning quality.

## Figures and Tables

**Table 1:** Comparative Summary of Robot Path Planning Approaches and Fuzzy Signatures.

Feature/Approach	Existing Fuzzy Path Planning (FPP) Methods	General Fuzzy Signature (FSigs) Applications	Proposed FSigs-Quadtree Approach
**Primary Data Structure**	Often uses linear rule-bases, sensor models, or dynamic windows [21,22].	Nested vectors or graphical FSigs focused on symbolic data [24,25,26].	Quadtree-organized hierarchical FSigs combined with $A^{*}$ path planning
**Main Application Focus**	Real-time control, local obstacle avoidance, trajectory generation [21,22,25,27].	Medical diagnosis, image processing, data mining, and knowledge representation [8,9,10,16].	Empirical study on aggregation operator impact for global path planning efficiency
**Hierarchy/Structure**	May utilize fuzzy logic, but lacks a formalized multidimensional hierarchy [9].	Hierarchical but non-spatial and focused on abstract symbolic relations [7,25,26].	Strictly coupled spatial (Quadtree) and fuzzy (FSigs) hierarchy optimized for rapid spatial queries and dimensionality consistency
**Research Gap Addressed**	Lack of systematic use of FSigs for environment mapping [24].	Lack of empirical analysis on aggregation operator effects on robot performance [28].	Provides the first systematic comparison of core aggregation operators (min, max, prod, mean) for FSigs in robot path planning

**Table 2:** Execution Time and Path Length results for each aggregation operator. The best values are indicated in **bold** and the second best values are in *italic*.

	Parameters	Goal Position			
		(−10, −10)	(10, −10)	(10, 10)	(−10, 10)
**MIN**	Path Length (nodes)	*27*	*30*	*24*	*23*
	Execution Time (s)	*0.1020*	*0.1237*	*0.0922*	0.1146
**MAX**	Path Length (nodes)	31	34	28	25
	Execution Time (s)	0.1923	0.1344	0.0967	0.1079
**PROD**	Path Length (nodes)	**26**	**27**	**22**	**22**
	Execution Time (s)	**0.0964**	**0.1111**	**0.0913**	**0.0961**
**MEAN**	Path Length (nodes)	30	*30*	*24*	*23*
	Execution Time (s)	0.1036	0.1446	0.0957	*0.0984*

## Data Availability

All data available regarding this study are shared in this paper.
